# Transcriptional response of individual Hawaiian *Culex quinquefasciatus* mosquitoes to the avian malaria parasite *Plasmodium relictum*

**DOI:** 10.1186/s12936-022-04271-x

**Published:** 2022-08-29

**Authors:** Francisco C. Ferreira, Elin Videvall, Christa M. Seidl, Nicole E. Wagner, A. Marm Kilpatrick, Robert C. Fleischer, Dina M. Fonseca

**Affiliations:** 1 Center for Conservation Genomics, Smithsonian Conservation Biology Institute, Washington, DC USA; 2 Center for Vector Biology, Entomology Department, Rutgers University, New Brunswick, NJ 08901 USA; 3 Department of Ecology, Evolution and Organismal Biology, Brown University, Providence, RI USA; 4 Institute at Brown for Environment and Society, Brown University, Providence, RI USA; 5 Animal Ecology, Department of Ecology and Genetics, Uppsala University, Uppsala, Sweden; 6 Department of Ecology and Evolutionary Biology, University of California, Santa Cruz, CA USA

**Keywords:** Differential gene expression, Transcriptome analysis, Vector, Invasive species, Vector-borne diseases

## Abstract

**Background:**

*Plasmodium* parasites that cause bird malaria occur in all continents except Antarctica and are primarily transmitted by mosquitoes in the genus *Culex*. *Culex quinquefasciatus*, the mosquito vector of avian malaria in Hawaiʻi, became established in the islands in the 1820s. While the deadly effects of malaria on endemic bird species have been documented for many decades, vector-parasite interactions in avian malaria systems are relatively understudied.

**Methods:**

To evaluate the gene expression response of mosquitoes exposed to a *Plasmodium* infection intensity known to occur naturally in Hawaiʻi, offspring of wild-collected Hawaiian *Cx. quinquefasciatus* were fed on a domestic canary infected with a fresh isolate of *Plasmodium relictum* GRW4 from a wild-caught Hawaiian honeycreeper. Control mosquitoes were fed on an uninfected canary. Transcriptomes of five infected and three uninfected individual mosquitoes were sequenced at each of three stages of the parasite life cycle: 24 h post feeding (hpf) during ookinete invasion; 5 days post feeding (dpf) when oocysts are developing; 10 dpf when sporozoites are released and invade the salivary glands.

**Results:**

Differential gene expression analyses showed that during ookinete invasion (24 hpf), genes related to oxidoreductase activity and galactose catabolism had lower expression levels in infected mosquitoes compared to controls. Oocyst development (5 dpf) was associated with reduced expression of a gene with a predicted innate immune function. At 10 dpf, infected mosquitoes had reduced expression levels of a serine protease inhibitor, and further studies should assess its role as a *Plasmodium* agonist in *C. quinquefasciatus*. Overall, the differential gene expression response of Hawaiian *Culex* exposed to a *Plasmodium* infection intensity known to occur naturally in Hawaiʻi was low, but more pronounced during ookinete invasion.

**Conclusions:**

This is the first analysis of the transcriptional responses of vectors to malaria parasites in non-mammalian systems. Interestingly, few similarities were found between the response of *Culex* infected with a bird *Plasmodium* and those reported in *Anopheles* infected with human *Plasmodium*. The relatively small transcriptional changes observed in mosquito genes related to immune response and nutrient metabolism support conclusions of low fitness costs often documented in experimental challenges of *Culex* with avian *Plasmodium*.

**Supplementary Information:**

The online version contains supplementary material available at 10.1186/s12936-022-04271-x.

## Background

*Plasmodium* parasites infect humans and a wide range of mammals, lizards, and birds globally [1, 2]. Avian malaria in Hawaiʻi, transmitted by invasive *Culex* mosquitoes and caused by *Plasmodium relictum,* another invasive species there, is an emblematic example of the deadly impacts a new parasite pose to wildlife [3, 4]. While the abundance and distribution of endemic Hawaiian honeycreepers (Fringillidae: Drepanidinae) have been dramatically impacted by habitat modification and the introduction of invasive vertebrates [5], avian malaria is one of the main causal factors behind the extinction or endangerment of several honeycreeper species [3, 4]. Avian malaria in Hawaiʻi is transmitted by the southern house mosquito (*Culex quinquefasciatus*), a highly competent vector [6, 7] that was introduced first to Lahaina, Maui presumably in 1826 [8]. It is now clear, however, that multiple *Cx. quinquefasciatus* introductions have occurred in Hawaiʻi [9] and the initial American-derived mosquitoes were later replaced by populations originating in the southwest Pacific region [10, 11], and this still is the only *Culex* species currently found in the Hawaiian Islands [9, 11, 12]. Although *Cx. quinquefasciatus* had been widespread across Hawaiʻi since the late 1800s [8], it was not until the 1950s that malaria was confirmed as a cause of mortality for native birds [3].

Low- and mid-elevation areas across Hawaiʻi provide habitat, temperature, and humidity favourable for both mosquito proliferation and *Plasmodium* development within the vector, effectively limiting honeycreeper populations mostly to cold high elevation habitats [13]. Most low-elevation native birds are susceptible to *Plasmodium* although some populations of Hawaiʻi ‘Amakihi (*Chlorodrepanis virens*) survive infections [14, 15] and persist in low-elevation areas [16]. Nonetheless, they can become infectious to mosquitoes [16, 17]. Consequently, higher mosquito infection rates are observed in areas with higher densities of native birds, suggesting that the presence of these species increases malaria transmission, which in turn may negatively affect the survival of other more susceptible native Hawaiian bird species in low elevation areas [17].

Reducing malaria transmission via suppression of mosquito populations and/or a reduction of vector competence of local *Cx. quinquefasciatus* could contribute to the reestablishment of Hawaiian bird populations to lowland areas, but mosquito control methods have been unsuccessful at protecting Hawaiian honeycreepers from malaria, partly because of difficult access to mosquito breeding sites and the effects of insecticides on non-target species [5]. Therefore, other methods to reduce or block *Plasmodium* transmission are needed [18]. One proposed strategy is the development of a synthetic gene drive system to make mosquitoes refractory or resistant to the parasite [19], and recent developments of CRISPR tools for *Cx. quinquefasciatus* [20] have opened exciting new opportunities for genetically engineered mosquitoes to be potentially released as a conservation option in the future. This approach, however, requires information about mosquito genes directly related to *P. relictum* invasion, development, and transmission.

*Plasmodium* parasites undergo a complex life cycle within their vectors, and the main developmental stages are relatively conserved among species that infect birds and mammals [21]. Blood feeding female mosquitoes ingest gametocytes, and these parasites develop into macrogametes and microgametes that fuse to form motile ookinetes as early as 16 h post mosquito feeding [4]. Ookinetes cross the blood meal peritrophic membrane and attach to the mosquito midgut epithelium, initiating the interaction between the parasite and its potential vector. Ookinetes then actively cross the midgut epithelia and continue their development. This migration is a critical bottleneck for *Plasmodium* development because an infected mosquito may activate immune-related genes to kill ookinetes [22]. Ookinetes that survive develop into oocysts where sporozoites are produced via sporogony using mosquito resources such as lipids, carbohydrates, and amino acids [23]. Mature oocysts burst and release thousands of sporozoites into the mosquito haemocoel, a subset of which reaches the salivary glands and cross its epithelial layer, where they remain until the mosquito takes another blood meal. During the probing process on the host, sporozoites in the mosquito saliva are injected into the skin where their journey continue in the vertebrate host [24].

Recent studies conducted on Hawaiian honeycreepers unveiled bird genes linked to immune functions that may be under selection in ‘amakihi populations exposed to malaria [15] and explored the transcriptome of *P. relictum* blood stages in experimentally infected ‘amakihis [25]. However, the genetic basis of the response of vectors to *Plasmodium* parasites in Hawaiʻi or in other geographical areas is still unknown, despite avian malaria being globally widespread [26]. Acquiring this information will allow the comparison of the responses of vectors of mammalian (*Anopheles* mosquitoes) and avian (*Culex* mosquitoes) *Plasmodium* parasites. These comparisons are particularly interesting given that the subfamilies Anophelinae and Culicinae diverged around 200 million years ago [27], while the split between avian and primate/rodent *Plasmodium* is estimated to have occurred around 45 million years ago [28]. Using avian malaria parasites and four mosquito genera as models, Huff [29] suggested that mosquito refractoriness to *Plasmodium* is driven by its immune response against the parasites. While his study forms the basis to our understanding of vector competence to malaria, the vector’s responses to *Plasmodium* have so far been studied mainly in human and rodent malaria systems using *Anopheles* mosquitoes.

Here, the transcriptional response of representative Hawaiian *Cx. quinquefasciatus* exposed to Hawaiian *P. relictum* was analysed. Mosquitoes were divided into two groups: one that fed on a *P. relictum*-infected canary (*Serinus canaria*), and another group that fed on an uninfected canary to serve as a baseline for analyses. *Plasmodium* parasites induce gene expression changes in *Anopheles* mosquitoes during ookinete invasion, oocyst development, and when sporozoites spread into the vector haemolymph and invade the salivary glands of the vector [22]. Some of these changes are measurable in the whole mosquito body even when parasite development is restricted to the midgut [30]. Therefore, the transcriptome of whole bodies of single *Cx. quinquefasciatus* was sequenced and quantified to address individual response to *P. relictum* at these three critical time points for *Plasmodium* development within the vector.

## Methods

### *Plasmodium relictum* isolation and *Culex quinquefasciatus* collection

*Plasmodium relictum* (lineage GRW4) was isolated from a single Hawaiʻi ‘amakihi (*Chlorodrepanis virens*) captured in Nanawale Forest Reserve (19°32′14.4"N 154°54′11.6"W) in February 2020. This is a low elevation area (98 m above sea level) where Hawaiʻi ‘amakihi constitute 20% of the avian community [17].

Around 100 µl of blood was collected from the brachial vein into a 1 ml syringe containing 14 µl of Citrate–phosphate-dextrose solution with adenine (Sigma-Aldrich, St. Louis, MO, USA), as described by Carlson et al. [31]. The sample was stored at 4º C for 48 h before being inoculated into the pectoral muscle of an uninfected domestic canary, *Serinus canaria*. Ten days post inoculation, this canary developed a parasitaemia (intensity of infection; see parasitaemia assessment below) of 1.72% and 100 µl of blood was collected as described above. That blood sample was shipped on ice from Hawaiʻi to Rutgers University in New Jersey and was intraperitoneally inoculated into two *Plasmodium*-free canaries 36 h after it had been collected.

To obtain the experimental mosquitoes, 30 *Culex quinquefasciatus* egg rafts were collected in Captain Cook, Hawaiʻi Island (19°27′40.6"N 155°53′47.4"W – 4.5 km from Kealakekua, described in Fonseca et al. [32]) at 204 m above sea level. The egg rafts were shipped to Rutgers University and maintained in an incubator at 26 ºC, with 70–80% relative air humidity under a photoperiod regime of 13: 11 h light/dark. After hatching, 250–300 larvae were kept in plastic pans (44 cm × 25 cm × 10 cm) provided with 0.2–0.4 g of ground fish food (Koi’s Choice^®^ Premium Fish Food) daily as described by Kauffman et al. [33]. Pupae were transferred to mosquito cages (30 cm^3^, BugDorm) kept in the same incubator, and emerging adults were maintained on a 10% sucrose solution. Female mosquitoes were fed on uninfected canaries to create an F1 generation of mosquitoes under laboratory conditions. Larvae originated from egg rafts laid by these mosquitoes were reared as described above and 300–400 pupae from different pans were pooled and then separated into two mosquito cages for the experimental challenges (see below). For all blood feeding procedures, canaries were immobilized in a plastic cylinder, in which only their legs are accessible to mosquitoes [34], and placed inside the mosquito cages maintained in the incubator. Experimental procedures were approved by Rutgers University Institutional Animal Care and Use Committee (PROTO201900075) and by University of California Santa Cruz IACUC (protocol kilpm2003). Permits for bird sampling include U.S. Department of the Interior Bird Banding Laboratory permit #23600, Hawaiʻi State Department of Land and Natural Resources Protected Wildlife Permit WL 19–23 Amend 01, Hawaiʻi State Access and Forest Reserve Special Use Permit. Mosquitoes and *Plasmodium* isolates were transported from Hawaiʻi to New Jersey under USDA-APHIS permits number 140413 and 141156.

### Mosquito infections and assessment of parasite development

Sixty to seventy female mosquitoes 7–8 days-old were allowed to feed for one hour on a single infected female canary (see above). This bird had been experimentally inoculated 18 days prior with a second-passage of *Plasmodium*, and was sampled every 2–3 days after the fifth day post infection. Blood samples were assessed by PCR [35] and light microscopy [2] for the presence of *Plasmodium*. For the latter, thin blood smears were prepared, fixed with absolute methanol, and stained with 10% Giemsa for 60 min. Parasitaemia was estimated as the number of parasites infecting 20,000 erythrocytes examined at high magnification (× 1000). Mosquitoes from the same batch as the ones that fed on the infected canary were allowed to feed on an uninfected female canary, also for 1 h, to serve as negative controls for baseline transcriptome data. Fully engorged mosquitoes from infected and control groups were transferred to separate cages and kept under the same conditions as described above. For transcriptome analyses, mosquitoes were processed at three time points of *P. relictum* development: (1) midgut invasion by ookinetes at 24 h post feeding (hpf); (2) oocyst development at 5 days post feeding (dpf); and (3) sporozoite release into the haemocoel and invasion of the salivary glands at 10 dpf. Previous experiments conducted under the same conditions revealed that ookinetes were present in the midgut 24 hpf; immature oocysts were present in the midgut at 5 dpf, but no sporozoites were detected in the salivary glands; mosquitoes at 8 dpf harboured sporozoites in the salivary glands and were already capable of infecting domestic canaries via blood feeding. Two extra incubation days were added at the last time point for the transcriptome experiments to allow for longer oocyst development and sporozoite invasion of salivary glands. At each timepoint, seven to nine mosquitoes from both infected and negative control groups were collected with a battery-operated aspirator and immediately transferred to an insulated box containing dry ice (for a total of 25 infected mosquitoes and 21 control mosquitoes). After this step, individual mosquitoes were quickly transferred to screw-cap microtubes on dry ice and were stored at −80º C until RNA extraction. Mosquito challenges were conducted in a USDA-APHIS inspected BSL-2 insectary at the Rutgers Center for Vector Biology.

### Mosquito dissection and parasite quantification

In addition to the 46 mosquitoes that were stored at − 80 °C, two additional mosquitoes from the infected group were dissected at each timepoint for *Plasmodium* evaluation. At 24 hpf, their midguts were dissected onto glass slides and the visible blood meal was homogenized with saline solution (0.9% NaCl). These preparations were air-dried, fixed with absolute methanol, and stained with 10% Giemsa solution for 60 min. Two mosquitoes were dissected at 5 dpf and at 10 dpf by individually pulling their midguts onto glass slides containing saline solution. A glass coverslip was gently placed over the midguts and subsequently examined with a microscope under 100 × and 400 × magnification to detect oocysts. Each midgut preparation was evaluated three times and there were no discrepancies among counts. After quantification of oocysts, each midgut was transferred to individual microtubes that were kept at -20º C. Using sterile dissecting needles, the salivary glands from these mosquitoes were extracted onto individual glass slides containing saline solution forming thin smears. Head and thorax remnants of dissected mosquitoes were transferred to individual microtubes and stored at -20º C. Slides with salivary glands preparations were air-dried, fixed with absolute methanol, and stained with 4% Giemsa solution for 60 min. DNA was extracted from the midguts and thorax remnants of mosquitoes dissected at 5 dpf and 10 dpf using DNeasy® Blood & Tissue Kit (QIAGEN, Hilden, Germany) following manufacturer’s instructions with the exception that the DNA was eluted in 50 μL of AE buffer. A PCR targeting the *Plasmodium cytb* locus [35] was used to test this material for the presence of *Plasmodium* and positive samples were sequenced to confirm parasite identity.

### RNA extraction, library preparation and sequencing

RNA from individual mosquitoes was extracted using TRIzol^®^ (Invitrogen, Carlsbad, CA, USA) followed by column purification using RNeasy mini kit^®^ (QIAGEN, Hilden, Germany). First, 500 μL of TRIzol^®^ and one 3-mm glass bead were added to the microtubes with mosquitoes and then the samples were homogenized in a TissueLyser^®^ (QIAGEN, Hilden, Germany) at 30 Hz for 3 min. An additional 600 μL of TRIzol^®^ was added to the samples that were incubated at room temperature for 3 min. After this step, 220 μL of chloroform was added, the tubes were shook by hand vigorously for 15 s and incubated at room temperature for 3 min. The samples were centrifuged at 12,000 g for 15 min in a cooled centrifuge and 650 μL of the upper aqueous phase was transferred to a new microtube. An equal volume (650 μL) of 70% ethanol was added to the supernatant and this mixture was transferred to Qiagen RNeasy^®^ mini columns (QIAGEN, Germantown, MD, USA). Downstream processes were performed following the manufacturer’s instructions with the additional DNase I digestion step. The RNA was suspended in 50 μL of RNAse-free water and checked using a NanoDrop™ 2000 (Thermo Scientific, Waltham, MA, USA).

The tubes containing the interphase and lower phenol–chloroform layers with TRIzol^®^ from infected mosquitoes were kept aside for DNA extraction. This material was transferred to new 1.5 μL microtubes containing 330 μL of 100% ethanol and downstream steps were conducted following the manufacturer’s protocol. These DNA samples were used to confirm via PCR (see above) that mosquitoes had acquired infection after feeding on the infected canary.

A total of 200 ng of RNA was used to prepare libraries from our 24 samples, which consisted in three control and five infected mosquitoes sampled at 24 hpf, 5 dpf and 10 dpf. mRNA was isolated using NEBNext Poly(A) mRNA Magnetic Isolation Module (New England Biolabs, Ipswich, MA, USA) following the manufacturer's protocol. The isolated mRNA was then used to make transcriptome libraries using the NEBNext Ultra II Directional RNA Library Prep Kit with NEBNext Multiplex Oligos for Illumina (Index Primers sets 1—4) according to the manufacturer's protocol. Library quality was assessed on an Agilent 2100 Bioanalyzer using High Sensitivity DNA reagents and chips (Agilent Technologies, Santa Clara, CA, USA). Library concentration was measured with a Qubit 2.0 Fluorometer using a dsDNA BR Assay Kit (Life Technologies, Carlsbad, CA, USA). All 48 libraries were pooled in an equimolar fashion and submitted to Genewiz (South Plainfield, NJ, USA) for paired-end sequencing (2 × 150 bp) in one lane of Illumina HiSeq 2500.

### Data analyses

Raw reads were trimmed of adaptors and quality-filtered using Trimmomatic (ver. 0.39 [36]). Read quality was assessed using FastQC (ver. 0.11.9; www.bioinformatics.babraham.ac.uk/projects/fastqc) together with MultiQC (ver. 1.9 [37]). A total of 286 million 150 bp paired-end reads passed quality control, with an average of 12 M reads per sample. HISAT2 (ver. 2.2.1 [38]) was used to map trimmed reads to the concatenated genomes of *Culex quinquefasciatus* (Johannesburg strain, ver. VectorBase 48; https://vectorbase.org/vectorbase/app), *Plasmodium relictum* (PrelictumSGS1-like/ PlasmoDB 48; https://plasmodb.org/plasmo/app/; [39]) and domestic canary (https://www.ncbi.nlm.nih.gov/assembly/GCF_007115625.1). The three genomes were concatenated to allow potential reads from mosquitoes, parasites, and birds to map to their respective genomes. In a second step, only the reads that mapped to the *Cx. quinquefasciatus* genome using Samtools (ver 1.10 [40]), were kept to ensure only the mosquito genes were analysed. The reads were then counted using HTSeq (ver. 0.11.1 [41]), and differential gene expression analyses were performed with DESeq2 (ver. 1.28.1 [42]) in R (ver. 4.0.3; R Development Core Team, 2020 [43]). Counts were normalized for library size differences using the geometric mean and modelled with a negative binomial distribution.﻿ To visualize samples on a Principal component analysis (PCA) plot without bias, Variance Stabilizing Transformation of counts was performed according to the manual. Because mosquito transcriptomes had been sequenced individually, and not pooled as in most studies, the biological variation between individuals could be assessed in these analyses. Differentially expressed genes (DEG) were compared between infected and control mosquitoes within each time point using Benjamini and Hochberg false discovery rate to correct for multiple testing. Genes were considered significantly differentially expressed using the default DESeq2 threshold of p-adjusted values < 0.1.

Gene Ontology (GO) analyses of the DEGs were conducted in VectorBase 54 [44] using the domains biological processes and molecular functions. Functional groups with a Benjamini–Hochberg false discovery rate (FDR) of < 0.1 were considered as statistically enriched. The list of enriched GO terms was filtered for redundant terms manually and through REVIGO [45]. The final list was used for visual representations as described by Bonnot et al. [46].

## Results

### *Plasmodium relictum *development in mosquitoes

The infected canary was *Plasmodium*-positive by both PCR and microscopy for the first time at 7 days post infection (dpi), displaying an initial parasitaemia of 0.02% and reaching a peak parasitaemia of 1.11% at 16 dpi. On the day of the mosquito exposure experiments (18 dpi), the bird had a parasitaemia of 0.92%, with 0.12% of the total erythrocytes infected with mature gametocytes (the sexual stages that give rise to male and female gametes in the mosquito midgut). This parasitaemia is known to occur naturally in Hawaiʻi [16].

No ookinetes were detected in the midguts of the two mosquitoes dissected at 24 hpf. At 5 dpf, one and two oocysts were identified in the midgut of each of the two mosquitoes dissected, and no sporozoites were found in the salivary glands of these specimens. Thorax remnants from both mosquitoes dissected at 5 dpf were PCR-negative, confirming that sporozoites were not present in the mosquitoes’ salivary glands at that time point. At 10 dpf, three oocysts were identified in the midgut preparations of both mosquitoes, as well as low densities of sporozoites in the salivary glands. Midguts from 5 and 10 dpf and thorax remnants at 10 dpf were PCR-positive and *cytb* sequencing confirmed the expected GRW4 lineage identity of *Plasmodium relictum* present in the infected mosquitoes. All mosquitoes survived the 10-day period of the experiment.

For the mosquitoes that were prepared for RNA extraction, *Plasmodium* infection was confirmed by PCR in all samples at 24 hpf and 10 dpf. One of the mosquitoes from the 5 dpf group was PCR-negative and was substituted with a backup mosquito that was positive by PCR. Therefore, infection was confirmed in all mosquitoes exposed to *Plasmodium* before transcriptome analyses.

### Overall Culex transcriptome response to* Plasmodium relictum*

**Fig. 1:**
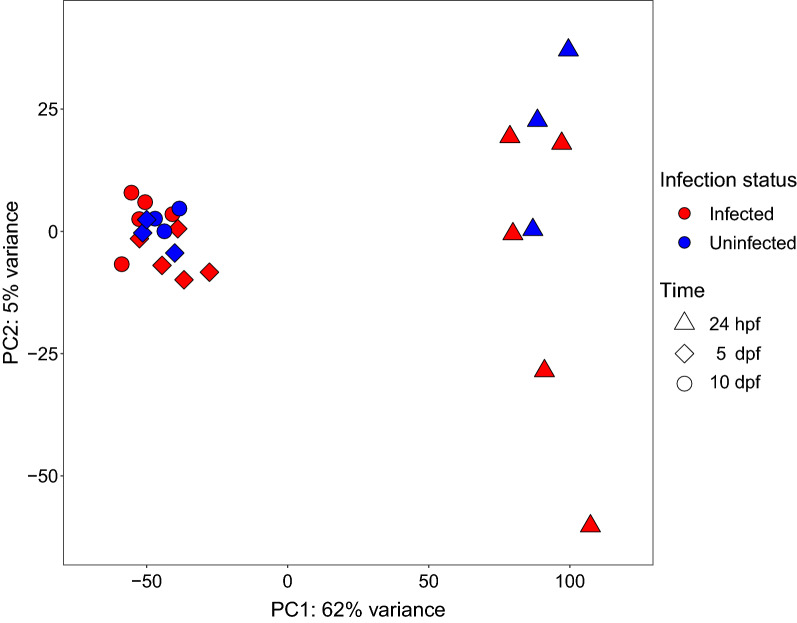
Principal component analysis (PCA) of transcriptome variation in *Culex quinquefasciatus* mosquitoes at three time point after feeding on either a negative-control canary (Uninfected) or a canary infected with a Hawaiian strain of *Plasmodium relictum* GRW4 (Infected). Time points analysed were: 24 h post feeding (24 hpf = ookinete invasion of mosquito midgut), 5 days post feeding (5 dpf = oocyst development), and 10 days post feeding (10 dpf = oocyst maturation, sporozoite release and invasion of salivary glands). Most of the variance was explained by mosquitoes 24 hpf regardless of *Plasmodium* infection status

Most of the transcriptome variation in the PCA (62%) was driven by the response of 24 h post feeding mosquitoes, regardless of their *Plasmodium* infection status (Fig. 1). There was no clear clustering of mosquitoes at 5 dpf or at 10 dpf or based on their infection status. This indicates a unique transcriptional response during blood digestion, broadly irrespective of the presence of the parasites. Two genes were differentially expressed at more than one time point: CPIJ018704 (Transmembrane protein 104) had higher expression levels at 5 dpf and lower expression levels at 10 dpf in infected mosquitoes compared to the control group; CPIJ010933 (uncharacterized protein) had lower expression levels at 5 dpf, but higher expression levels at 10 dpf.

### Differential transcriptional response at 24 h post blood feeding—ookinete invasion

**Fig. 2:**
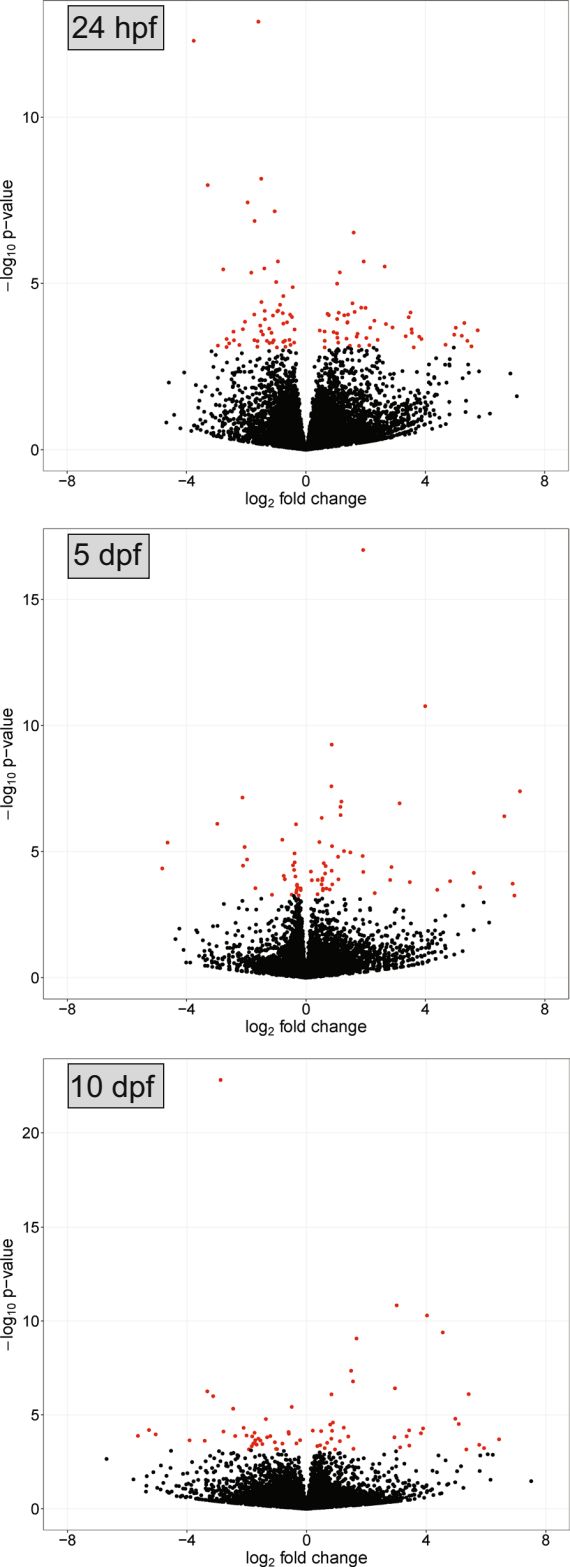
Volcano plots showing *Culex quinquefasciatus* gene expression at three time points after feeding on a canary infected with a Hawaiian strain of *Plasmodium relictum.* Mosquitoes that fed on an uninfected canary were used as negative controls, and time points analysed are described in the legend of Fig. 1. Red circles illustrate significant genes with lower (negative) or higher (positive) expression levels in infected mosquitoes compared to uninfected ones. Black circles illustrate nonsignificantly expressed genes

**Fig. 3:**
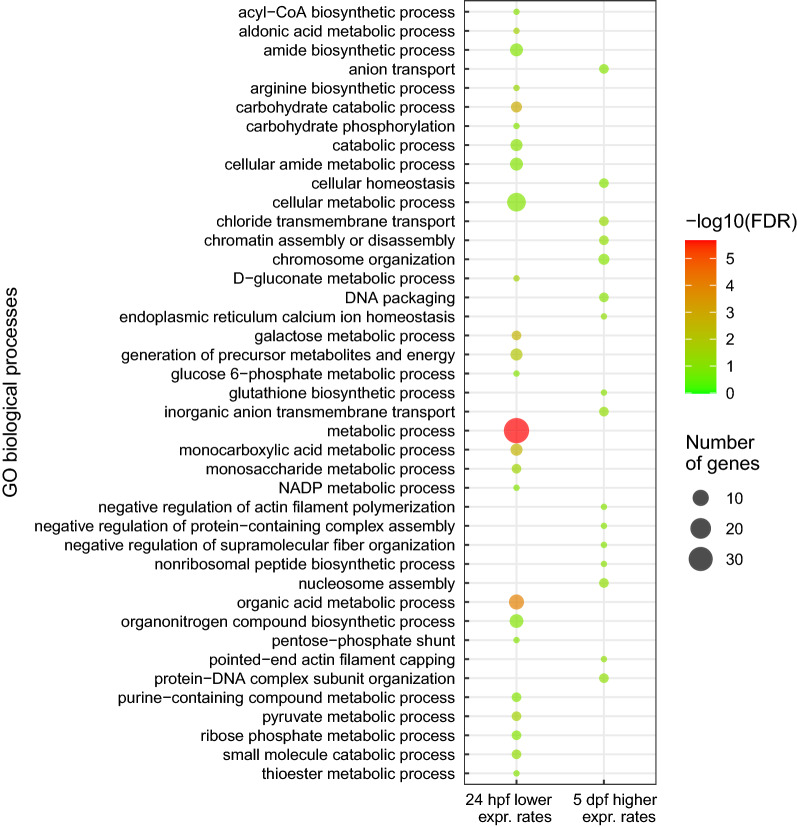
Enriched Gene Ontology terms for biological processes among differentially expressed genes in infected mosquitoes compared to uninfected ones. Only time points with statistically significant enriched GO terms are displayed. hpf = hours post feeding, dpf = days post feeding

During the initial *Plasmodium* invasion, 24 h after feeding on an infected bird, 109 genes were differentially expressed in the infected mosquitoes compared to the control group, with 53 genes having higher expression levels, and 56 genes having lower expression levels (Fig. 2, Additional file 2: Table S1). Infected mosquitoes had higher expression levels of genes related to cytoskeleton organization (CPIJ001347, CPIJ003238 and CPIJ013059) and of a Down syndrome cell adhesion molecule gene paralog (CPIJ007041). Among the most significant genes with lower expression rates in infected mosquitoes, functions included peptidase activity (CPIJ006867 and CPIJ019883), zinc ion binding (CPIJ013381) and carbohydrate binding (CPIJ015611). Gene ontology analyses using genes with lower expression levels revealed enriched biological processes that include galactose catabolism and acetyl-CoA biosynthesis (Fig. 3). Enriched molecular processes included oxidoreductase activity, serine-type endopeptidase activity and ion binding for a variety of metals. Almost significant (FDR = 0.107) enriched GO terms of genes with higher expression levels in infected mosquitoes were related to molecular functions such as calcium transportation, cobalamin binding and peptidoglycan muralytic activity (Additional file 3: Table S2, Additional file 1: Fig. S1).

### Differential transcriptional response at 5 days post blood feeding—oocyst development

During oocyst development at 5 days dpf, before sporozoites are released into the mosquito haemocoel, 45 genes had higher expression levels and 27 genes had lower expression levels in infected mosquitoes compared to the uninfected control mosquitoes (Fig. 2, Additional file 4: Table S3). Six out the 10 most significant differently expressed genes with higher and with lower expression levels in infected mosquitoes have unknown functions. However, the gene CPIJ007162, which contains a predicted N-acetylmuramoyl-L-alanine amidase activity domain with innate immune function had four times lower expression levels in infected mosquitoes compared to the uninfected ones. Molecular functions enriched in genes with lower expression levels were related to carbamoyl-phosphate synthase and 4-alpha-hydroxytetrahydrobiopterin dehydratase activities (Additional file 1: Fig. S1). At this time point, GO terms associated with biological processes such as chloride transmembrane transport, chromatin organization and DNA packaging were enriched for genes with higher expression levels (Fig. 3, Additional file 5: Table S4).

### Differential transcriptional response at 10 days post blood feeding—sporozoite migration and invasion of salivary glands

At 10 dpf, after oocyst maturation and release of sporozoites as well as salivary glands invasion, 36 genes had lower expression levels and 37 genes had higher expression levels in infected mosquitoes (Fig. 2, Additional file 6: Table S5). An adhesive serine protease (CPIJ007535), a serine protease inhibitor (Serpin B8, CPIJ017784) and an uncharacterized gene (CPIJ009078) related to chitin metabolism had low expression levels at this time point, while a glutamate dehydrogenase gene (CPIJ014605) and six nucleotide binding genes had higher expression levels in the infected mosquitoes. GO terms of molecular functions associated with ribonucleotide and GTP binding as well as oxidoreductase activity were enriched for genes with higher expression levels (Additional file 7: Table S6, Additional file 1: Fig. S1). No GO terms were significantly enriched for genes with lower expression in infected mosquitoes.

## Discussion

This is the first study to show mosquito transcriptome responses to an avian malaria parasite. Here, natural conditions were reproduced as much as possible: 1) single wild-caught mosquitoes with minimal laboratory colonization (F1) were used, 2) a “fresh” Hawaiian parasite isolate (only two passages in canaries) was obtained for these experiments, 3) and a donor bird with parasite intensity levels that *Culex* mosquitoes may be exposed to in Hawaiʻi was used (16). These attributes suggest that the results showed reflect real-world scenarios of *Culex*-*Plasmodium* interactions happening in Hawaiʻi. Serial parasite passages increase infection burden in birds and correlate with higher oocyst burden and longer lifespan in mosquitoes (47), which are artificial effects avoided in this experiment. By simulating natural conditions while still benefiting from the controlled aspects of laboratory experiments, this approach allowed the evaluation  of *P. relictum* effects on Hawaiian *Cx. quinquefasciatus*. The pairwise comparisons within each time point revealed that *Culex* transcriptional responses to *Plasmodium* infection were more pronounced during the early stages of parasite invasion than during development and sporozoite invasion of the salivary glands. This pattern is similar to the generally strong immune response mounted by *Anopheles* mosquitoes during *Plasmodium* invasion of the midgut epithelia [48].

Only two genes were differently expressed at more than one time point, suggesting that the *Culex* transcriptional response to *Plasmodium* is different across infection stages by utilizing different genes. Interestingly, a higher proportion of *P. relictum* differently expressed genes are shared across different developmental stages in the vector [49]. However, GO enrichment showed that biological processes and molecular functions orchestrated by *P. relictum* [49] and by *Cx. quinquefasciatus* differently expressed genes (Fig. 3 and Additional file 1: Fig. S1) are more specific according to parasite developmental stage, revealing that mosquito transcriptional response to infection may be coupled with the transcriptional changes in invading parasites.

Three genes involved in cytoskeleton organization had four times higher expression in infected mosquitoes at 24 hpf and no genes related to this biological process had lower expression rates at the same time point. Ookinete invasion of the midgut epithelia activates cytoskeleton reorganization in *Anopheles* [50], indicating this is a common feature of mosquito response to avian and mammalian *Plasmodium* during early stages of infection. Four genes with functions predicted to be involved in calcium transportation or binding had higher expression levels in infected mosquitoes at 24 hpf. Calcium is essential for ookinete motility [51] and these alterations may supply parasites with Ca^2+^ which may facilitate midgut invasion, warranting future studies to investigate whether *Plasmodium* parasites directly alter calcium concentration in midgut epithelial cells. In *Anopheles* mosquitoes, increased nitric oxide synthase activity reduces parasite invasion after feeding on infected hosts [52], and gene transcription can increase as early as 6 hpf [53]. Oxidoreductase activity was enriched in infected mosquitoes at 24 hpf, encompassing 12 genes with lower expression rates in infected mosquitoes. However, it remains to be determined whether this decreased activity would reduce the production of reactive nitrogen compounds that cause damage to *Plasmodium* parasites at early stages of infection [52]. One of the three Down syndrome cell adhesion molecule gene paralogs had higher expression levels during ookinete invasion. This molecule mediates *Plasmodium* inhibition in *Anopheles* during ookinete migration through the midgut epithelia, before the formation of oocysts [54] and results shown here indicate that it may likewise participate in *Culex* response to avian malaria parasites.

At 24 hpf, genes related to apoptosis and cell immunity that are known to be overactivated during *Plasmodium* invasion in *Anopheles* were not differently expressed even though this stage of infection is marked by strong physiological and immune responses to infection [22]. This may be because the host bird used was harbouring a parasite intensity that translated to low numbers of parasites detected in the mosquitoes that were dissected. Bird transcriptome response to avian malaria is highly dependent on blood parasite load (parasitaemia), which may be related to pathogenicity [55]. Also, Videvall et al. [55] found that low levels of circulating parasites were associated with a reduced transcriptome response to infection when compared with higher parasitaemia. Further studies using vectors exposed to birds harbouring different levels of parasitaemia may clarify if mosquito response at the transcriptome level is proportional to the number of parasites undergoing development in the vector. Another reason for the absence of changes in the expression of these genes might be because the *Cx. quinquefasciatus* genome is not as well assembled and annotated as those of *Anopheles gambiae* and *Aedes aegypti* [44]. In this study, 14.5% of *Cx. quinquefasciatus* DEGs in response to *P. relictum* infection were annotated as uncharacterized proteins or unspecified products with no predicted function, which limited the interpretation of some of the results.

It has been observed that at the start and early acceleration of the sporogonic division, oocysts seem to be refractory to the mosquito immune response [56], and this may explain the reduced transcriptome response to *Plasmodium* infection observed at 5 dpf. However, some studies showed that mosquito immune response can destroy young oocysts because total numbers decrease during parasite development in *Anopheles* [22]. The number of oocysts of avian *Plasmodium* also decrease during oocyst maturation in *Culex* [49], and this may be due to immune-mediated parasite killing. An unspecified gene with a predicted peptidoglycan binding function (CPIJ007162) was downregulated at 5 dpf. Peptidoglycan recognition proteins (PGRP) are important in the regulation of the Immunodeficiency (Imd) pathway in insect midguts, a pathway shown to kill *Plasmodium* in *Anopheles* midguts (57). Some PGRP function as *P. falciparum* antagonists, reducing prevalence in *Anopheles coluzzii* via Imd activation, while other PGRP genes from this family promote tolerance to *Plasmodium* by a downregulation of systemic Imd [58]. This is the only gene differently expressed in this study that has a predicted innate immune function, and it cannot be inferred whether the downregulation of this gene would affect *P. relictum* development within infected mosquitoes. Therefore, immune pathways in vector response to *Plasmodium* oocyst development remain to be described in *Culex* mosquitoes.

The mild transcriptome changes at 10 dpf indicate that a relatively small response is elicited in *Culex* to sporozoites during haemocoel migration and invasion of the salivary glands. This may be because salivary gland invasion by *Plasmodium* parasites generally induces lower mosquito physiological responses when compared to ookinete invasion of the midgut [59]. In *Anopheles* mosquitoes, some serine protease inhibitors, such as SRPN2, are known to facilitate *Plasmodium* midgut invasion and survival [60]. SRPN6 is upregulated in the salivary glands of infected mosquitoes [61] and reduce the number of invading sporozoites [62]. In contrast, SRPN B8 (CPIJ017784) had lower expression levels in infected mosquitoes at 10 dpf, and future studies are warranted to investigate potential agonistic effects between this gene and *P. relictum* in *Culex.*

More than 80% of sporozoites released in the haemolymph are quickly destroyed before they invade the salivary glands [63]. Drivers of mosquito response to circulating parasites are still unknown, and this study did not find specific immune-related genes that could be involved in sporozoite elimination by *Culex* mosquitoes. High nitric oxide (NO) concentrations driven by increased nitric oxide synthase (NOS) expression reduce *Plasmodium* development at initial stages in the midgut [53]. Expression levels of this gene are increased in the whole body of mosquitoes at the initiation of sporozoite release [52], but are not affected [61] or can be reduced in the salivary glands during *Plasmodium* invasion [64]. A glutamate dehydrogenase gene, which has a predicted oxidoreductase function, had higher expression levels in infected *Culex* at 10 dpf, but it is not possible to infer if these changes translate into increased NO production in the body or salivary glands.

Although parasite invasion (24 hpf) was associated with a reduction in galactose catabolism and acetyl-CoA biosynthesis, parasite development (5 dpf and 10 dpf), did not elicit strong changes in the expression of gene groups involved in vector nutrient metabolism, which could be related to the low parasite numbers detected in the infected mosquitoes. Such changes are likely to be more evident in vectors with high parasite loads if disruption of mosquito nutrient metabolism is proportional to parasite burden. Rodent malaria parasites trigger immune responses in *Anopheles* that reduce fitness and survival, while *P. falciparum* suppresses these responses, preventing the physiological cost of infection (reviewed by Shaw et al. [23]. Studies using a diversity of avian malaria parasites and bird-biting *Culex* mosquitoes found that *Plasmodium* infection does not reduce vector survival [65, 66], or may presumably increase it [66, 67] at the expense of reduced fecundity [67], although infection may reduce the life-span of nutritionally-stressed mosquitoes [68]. These findings combined with the results shown here suggest that coevolution in some avian malaria transmission systems may have led to associations of low virulence and fitness costs to the vectors.

## Conclusions

This first study to analyze transcriptional responses of malaria vectors in non-mammalian systems revealed a relatively minor gene expression response in *Culex* during *Plasmodium* infection. Because the experimental conditions used mimicked those in nature (single mosquitoes with minimal laboratory inbreeding, few parasite passages, and parasite loads common in nature), the results presented here provide important baseline information about transcriptional responses in avian *Plasmodium* vectors at different stages during parasite development. The small changes in the expression of genes related to nutrition metabolism and immune response indicate that the costs of infection for the vector may be minimal in the Hawaiian malaria system. The experimental set up involved a single infected bird donor to reduce biological and environmental variation. For that reason, future studies using different vector-parasite combinations and exposing mosquitoes to infected birds harbouring different *Plasmodium* intensities should be conducted to pinpoint genes likely to be associated with vector resistance or tolerance to avian *Plasmodium* infection.

## Supplementary Information

**Additional file 1: Figure S1.** Enriched Gene Ontology terms for molecular functions among differentially expressed genes in infected mosquitoes compared to uninfected ones. GO terms for genes with higher expression rates in infected mosquitoes at 24 h post-feeding were almost significantly enriched (FDR = 0.107) and are displayed. No GO terms were enriched for genes with lower expression in infected mosquitoes at 10 days post feeding. hpf = hours post feeding, dpf = days post feeding.

**Additional file 2: Table S1.** Genes differentially expressed in Culex quinquefasciatus 24 h post feeding on a canary infected with Plasmodium relictum compared to mosquitoes that fed on an uninfected bird. Sets are separated into genes that had higher and lower expression rates in the infected group. Gene product description and function information was retrieved from VectoBase (https://vectorbase.org/vectorbase/app).

**Additional file 3: Table S2.** Significantly enriched Gene Ontology terms among differently expressed genes in Culex quinquefasciatus 24 h post feeding on a canary infected with Plasmodium relictum compared to mosquitoes that fed on an uninfected canary. Sets are separated into genes that had higher and lower expression rates in the infected group. We analyzed biological processes and molecular function GO terms in VectorBase (https://vectorbase.org/vectorbase/app).

**Additional file 4: Table S3.** Genes differentially expressed in Culex quinquefasciatus 5 d post feeding on a canary infected with Plasmodium relictum compared to mosquitoes that fed on an uninfected bird. Sets are separated into genes that had higher and lower expression rates in the infected group. Gene product description and function information was retrieved from VectoBase (https://vectorbase.org/vectorbase/app).

**Additional file 5: Table S4.** Significantly enriched Gene Ontology terms among differently expressed genes in Culex quinquefasciatus 5 d post feeding on a canary infected with Plasmodium.

**Additional file 6: Table S5.** Genes differentially expressed in Culex quinquefasciatus 10 d post feeding on a canary infected with Plasmodium relictum compared to mosquitoes that fed on an uninfected bird. Sets are separated into genes that had higher and lower expression rates in the infected group. Gene product description and function information was retrieved from VectoBase (https://vectorbase.org/vectorbase/app).

**Additional file 7: Table S6.** Significantly enriched Gene Ontology for molecular function among genes that had higher expression rates in Culex quinquefasciatus 10 d post feeding on a canary infected with Plasmodium relictum compared to mosquitoes that fed on an uninfected canary. Analyses were conducted in VectorBase (https://vectorbase.org/vectorbase/app).

## Data Availability

Supporting information will be available online. Sequences have been uploaded to the Sequence Read Archive (SRA) at NCBI under accession number: PRJNA779986.
