# Supplementary figures and images for: Transcriptional response of individual Hawaiian Culex quinquefasciatus mosquitoes to the avian malaria parasite Plasmodium relictum
Source: Malar J. 2022 Aug 29;21:249. doi: 10.1186/s12936-022-04271-x (PMC9422152; doi:10.1186/s12936-022-04271-x)

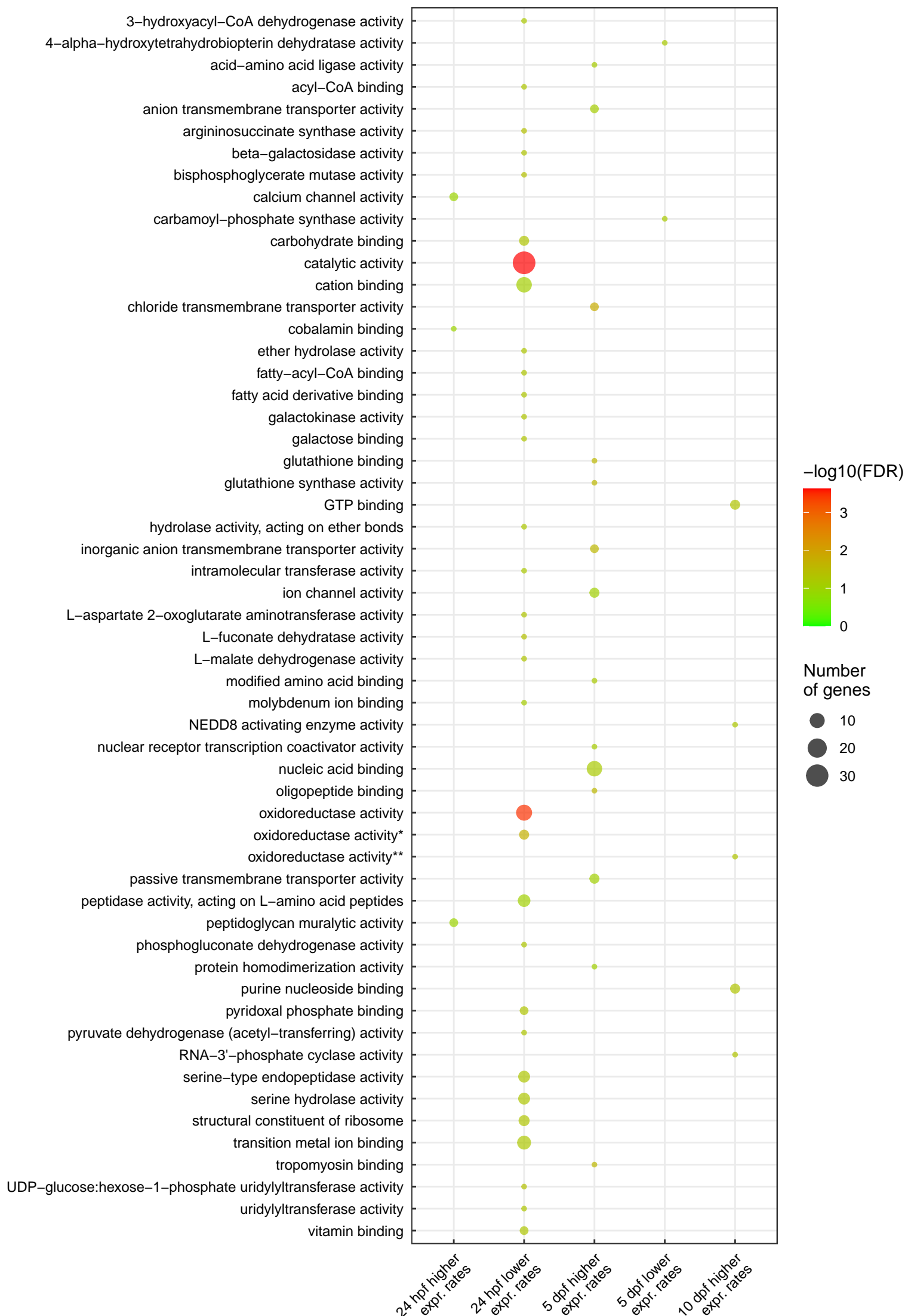

Supplement: Supplementary file 1 — Additional file 1: Figure S1. Enriched Gene Ontology terms for molecular functions among differentially expressed genes in infected mosquitoes compared to uninfected ones. GO terms for genes with higher expression rates in infected mosquitoes at 24 h post-feeding were almost significantly enriched (FDR = 0.107) and are displayed. No GO terms were enriched for genes with lower expression in infected mosquitoes at 10 days post feeding. hpf = hours post feeding, dpf = days post feeding. [file 12936_2022_4271_MOESM1_ESM.pdf]
